# Identification of Basement Membrane-Related Biomarkers in the Progression of Cutaneous Squamous Cell Carcinoma

**DOI:** 10.3390/ijms27031394

**Published:** 2026-01-30

**Authors:** Shuaijun Zou, Sijia Huang, Jun Liu, Ruiqian Yao, Xiaoyan Yang, Haixia Zhao, Lin Du, Liangzhe Wang, Yuanjie Zhu

**Affiliations:** 1 Department of Dermatology, Naval Medical Centre, Naval Medical University, Shanghai 200052, China; smmuzsj@163.com (S.Z.); yaorq999@163.com (R.Y.); xiaoyanyh@163.com (X.Y.); 18101823621@163.com (H.Z.); lynnie_du@126.com (L.D.); 2 School of Medicine, Shanghai University, Shanghai 200444, China; sijia-huang@shu.edu.cn; 3 Department of Naval Diving Medicine, Naval Medical Centre, Naval Medical University, Shanghai 200052, China; liujun4531@126.com

**Keywords:** basement membrane-related genes, cutaneous squamous cell carcinoma, Bowen’s disease, actinic keratosis, biomarkers

## Abstract

Basement membrane (BM) breaching is a critical hallmark of cutaneous squamous cell carcinoma (cSCC) invasion. This study aimed to identify novel BM-related genes (BMRGs) to effectively distinguish invasive cSCC from actinic keratosis (AK) and Bowen’s disease (BD), and to identify potential therapeutic targets. Single-cell RNA sequencing was used for BMRGs identification within keratinocytes and fibroblasts clusters. Protein–protein interaction network analysis and Lasso regression were performed for hub BMRGs screening, together with nomogram model construction and validation. In this study, 6–9 central hub BMRGs were identified for each stage during cSCC progression with a good AUC value (>0.8). In keratinocytes, BMRGs such as integrins (*ITGB1*, *ITGA3*, *ITGA6*), laminins (*LAMA3*, *LAMC1*), *CD44*, and *FN1* were upregulated in cSCC compared to AK or BD (adjusted *p* < 0.05); in fibroblasts, BMRGs including *ITGB1*, *ITGAV*, *LUM*, *BGN*, *SDC1*, and *FN1* were upregulated in cSCC (adjusted *p* < 0.05), suggesting their collective role in BM breaching and invasion, as well as a higher risk of BD. This study provides novel biological insights into the differentiation of progression pathways from AK or BD to cSCC, as well as potential targets for therapeutic intervention.

## 1. Introduction

Skin malignancies predominantly originate in epithelial tissues, with the stepwise transformation from pre-invasive lesions, such as actinic keratosis (AK) and Bowen’s disease (BD), to invasive neoplasia, such as cutaneous squamous cell carcinoma (cocci), involving sequential dysregulation of biological networks that govern essential functions of epithelial homeostasis [1]. The frequency of cSCC progression ranges from 0.025% to 16% per AK or BD lesion per year [2]. cSCC is the second-most common epithelial malignancy, with an annual incidence of more than 1 million in the USA [3,4] and the highest mutational burden of all solid tumors [5]. A plethora of risk genes, dominated by UV-induced sun damage, drive disease progression in conjunction with stromal interactions and local immunomodulation, thereby enabling continuous tumor growth [6,7]. To date, several gene profiles have been developed for the prediction of AK, BD, or cSCC, yet efficient biomarkers for distinguishing progression from AK or BD to cSCC remain elusive [8,9,10,11].

Emerging biological evidence supports the hypothesis that the invasive cSCC arises from two distinct pathways: progressing AK and BD [12]. On the one hand, AKs are lesions of epidermal keratinocytic dysplasia that result from chronic sun exposure and possess the potential to directly progress to invasive carcinoma. However, there is clinical disagreement regarding whether AKs should be classified as pre-invasive lesions, superficial SCC in situ or epiphenomena of chronically sun-damaged skin [13]. Previous studies have reported similar mutational spectra in AK and cSCC, and they have also confirmed several genes that are similarly altered in AK and cSCC [10]. So, it is necessary to identify genomic biomarkers driving progression from AK to malignant lesions. On the other hand, BD is another cause of frequent pre-invasive skin lesions and the most common precursors of invasive cSCC. The mechanisms of invasion and metastasis from BD to cSCC are complex and still not fully understood [14]. Studies have revealed that AK or BD lesions frequently harbor *TP53* or *NOTCH1* mutations, and additional somatic mutations and copy number alterations may lead to cSCC progression [15], which are common oncogenes leading to increased genomic instability and loss of cell cycle control. However, these findings are awkward to explain regarding the main issue of transition from pre-invasive lesions to cSCC, namely basement membrane (BM) breaching. Therefore, specific genomic biomarkers remain to be elucidated to characterize the progression of cSCC.

The extracellular matrix (ECM) plays a core role in the cSCC microenvironment [16]. As a barrier within the ECM, the BM is a cell-adherent extracellular matrix that is widely distributed in metazoan tissues, lies beneath epithelial cells, and surrounds most tissues, restricting the distant dissemination of cancer cells [17,18]. Epithelial–mesenchymal transition of the BM converts tumor cells from an epithelial phenotype to a mesenchymal-like phenotype and enables lymphovascular metastasis [19]. In addition, invasion through the BM is facilitated by remodeling and degradation of the ECM in conjunction with cancer-associated fibroblasts (CAFs) and matrix metalloproteinases (MMPs). CAFs can remodel the ECM by exerting pulling forces and MMPs enzymatically degrade the ECM, which together provide tracks for the cancer cells to migrate along [20]. Due to the significant role of BM in cancer metastasis, it has the potential to be a powerful predictor and antitumor target [21,22,23]. Recently, BM-related genes (BMRGs) have been the focus of research with a defined network of 222 BMRGs, where defects in BM protein expression and turnover were related to the occurrence of cancer [24]. Various prognostic BM-related indexes (BM score) have been constructed based on BMRGs for cancer prognosis, including breast cancer, lung adenocarcinoma, and liver cancer, etc. [21,22,23]. However, potential BM-related biomarkers in the progression of cSCC from pre-invasive lesions have yet to be developed.

In this study, single-cell RNA sequencing (scRNA-seq) was performed to identify the population of cells in AK, BD, and cSCC according to the biomarkers. Since keratinocytes and fibroblasts are the main epidermic and mesenchymal cells involved in tumor invasion and progression, these two cell groups were selected as representatives to identify the significant biomarkers [25,26,27]. Differentially expressed BMRGs, functional enrichment analysis, hub gene identification, and prognostic model construction were studied in order to fully understand the impact of BMRGs on the transformation from AK or BD to cSCC. This study aimed to explore the molecular mechanism between BM and the progression of cSCC, as well as on invasion, metastasis, and personalized treatment for different stages of lesions in cSCC patients.

## 2. Results

### 2.1. Differentially Expressed BMRGs

**Figure 1:**
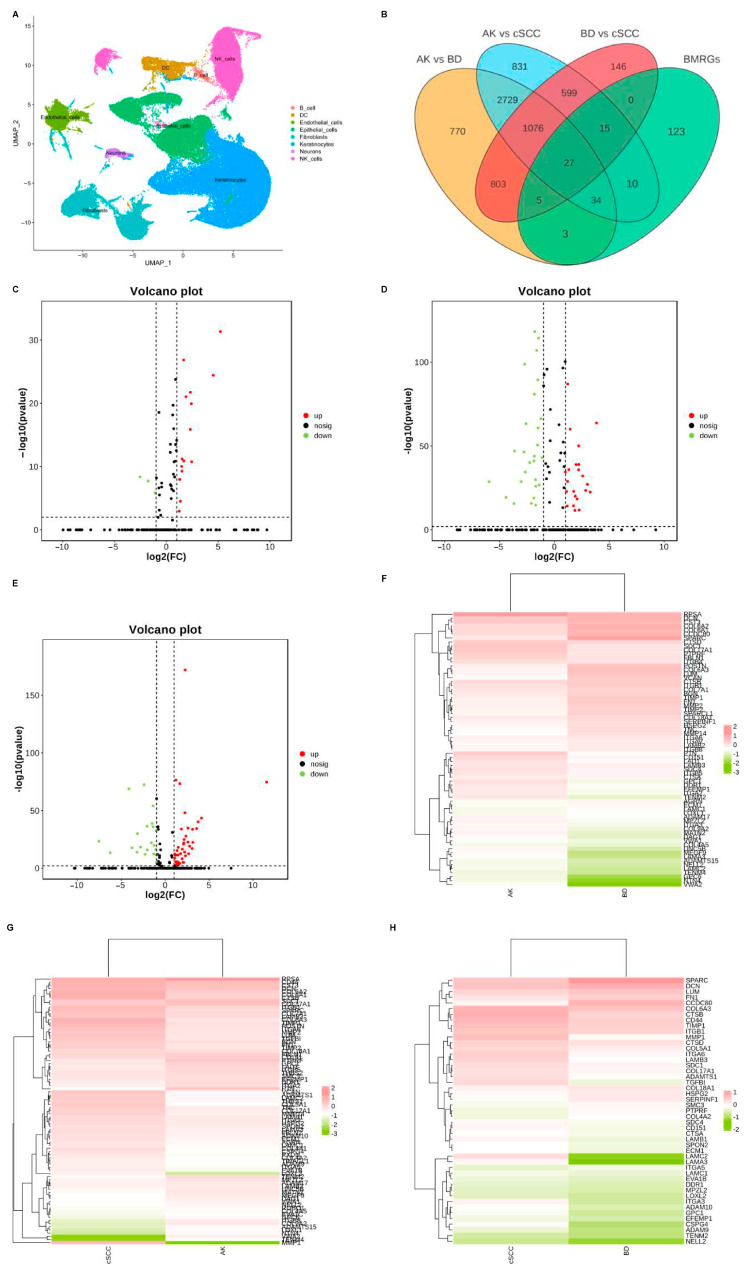
Cell clustering and differentially expressed BMRGs of keratinocyte clusters. (**A**) UMAP plot for visualization of cell clusters; (**B**) Venn diagram showing DEGs and differentially expressed BMRGs in keratinocyte clusters; volcano plots of BMRGs in (**C**) AK vs. BD, (**D**) AK vs. cSCC, and (**E**) BD vs. cSCC; and heatmaps of BMRGs in (**F**) AK vs. BD, (**G**) AK vs. cSCC, and (**H**) BD vs. cSCC.

Dimensionality reduction was achieved using principal component analysis (PCA), and the uniform manifold approximation and projection (UMAP) technique was employed to visualize single-cell populations (Figure 1A). Subsequently, the DEGs between each group of keratinocyte and fibroblast populations were analyzed (|logFC| > 0.50, adjusted *p* < 0.05), and differentially expressed BMRGs were selected via a Venn plot. For keratinocyte clusters, 69, 86, and 47 significantly differentially expressed BMRGs were found in AK vs. BD, AK vs. cSCC, and BD vs. cSCC, respectively (Figure 1B–H). Similarly, 70, 86, and 73 differentially expressed BMRGs were identified in AK vs. BD, AK vs. cSCC, and BD vs. cSCC among fibroblast clusters, all of which were shown via heat maps and volcano plots (Figure S1).

### 2.2. Functional Enrichment Analysis of Differentially Expressed BMRGs

**Figure 2:**
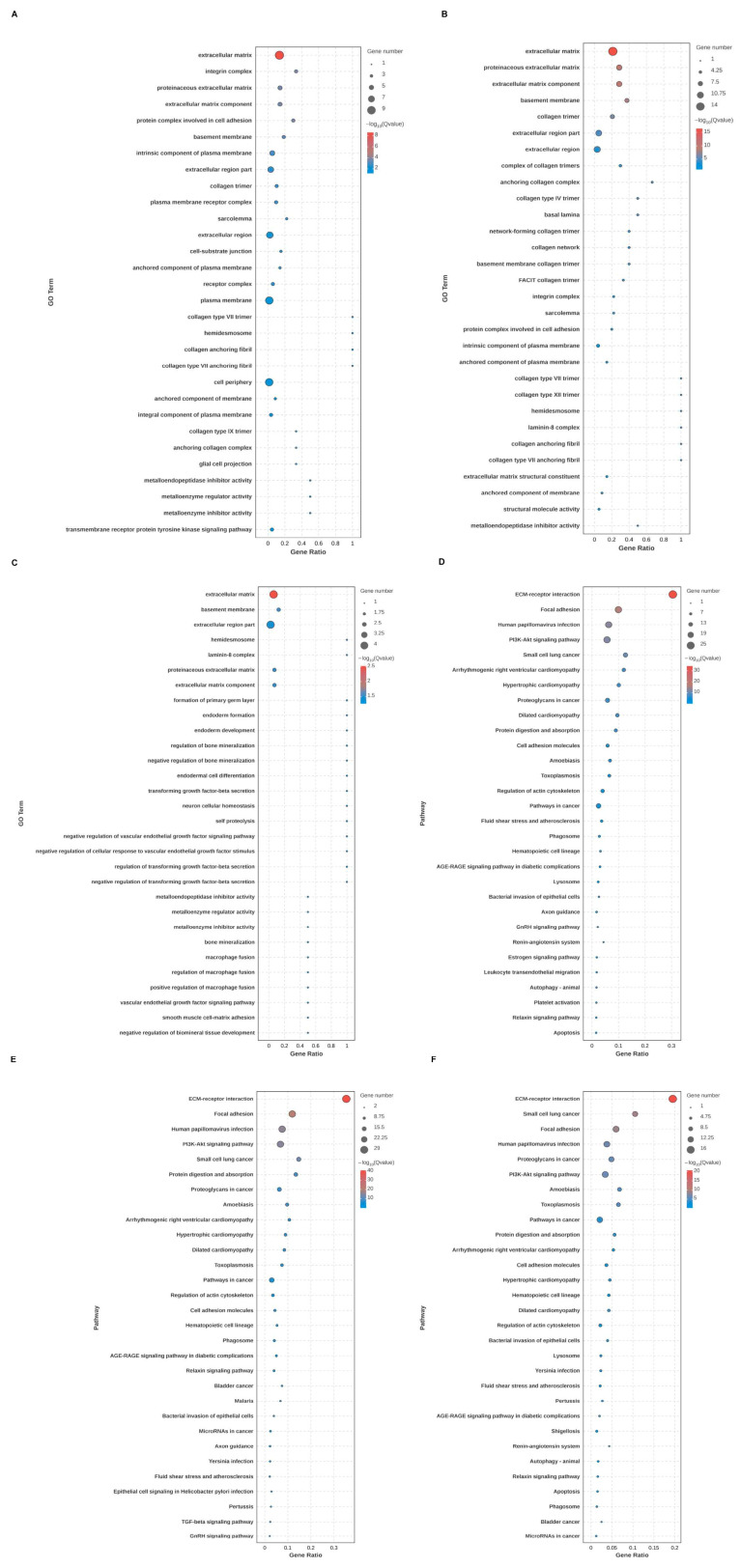
Functional enrichment analysis of keratinocyte clusters. (**A**,**B**) Bubble chart of GO enrichment analysis and KEGG enrichment analysis of AK vs. BD with top 30 functions or pathways listed. (**C**,**D**) Bubble chart of GO enrichment analysis and KEGG enrichment analysis of AK vs. cSCC with top 30 functions or pathways listed. (**E**,**F**) Bubble chart of GO enrichment analysis and KEGG enrichment analysis of BD vs. cSCC with top 30 functions or pathways listed.

Through GO and KEGG analysis, the biological function, cellular localization, and molecular function of DEGs of BMRGs in various groups were found. Between each group of keratinocytes and fibroblasts, it can be concluded that the cell localization of the differentially expressed BMRGs were primarily located in the extracellular matrix, proteinaceous extracellular matrix, and basement membrane, with molecular functions centered on the structural components of the extracellular matrix. KEGG analyses indicated that the functional pathways of differentially expressed genes were primarily concentrated on ECM-receptor interactions, focal adhesions, and PI3K-Akt signaling pathways (Figure 2A–F and Figure S2). Overall, these findings highlighted biological processes and signaling pathways linked to notable differences in BMRGs across different lesion stages.

### 2.3. Hub BMRGs Screening

**Figure 3:**
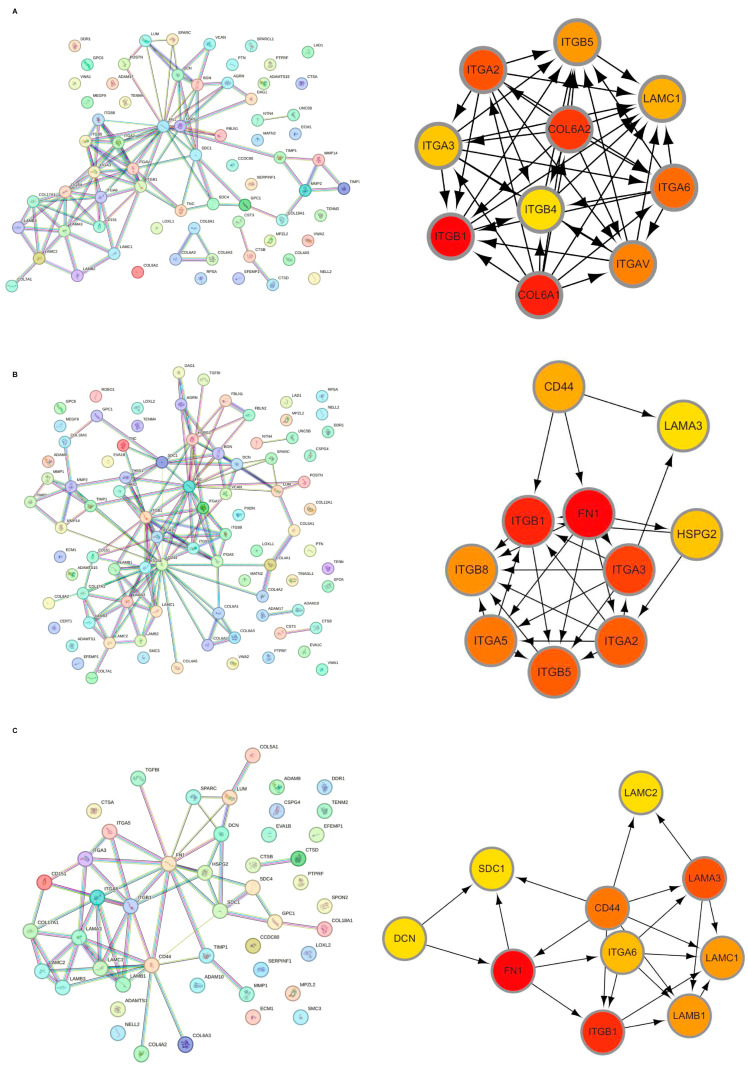
Hub BMRGs screening of keratinocyte clusters. PPI network construction of differentially expressed BMRGs in (**A**) AK vs. BD, (**B**) AK vs. cSCC, and (**C**) BD vs. cSCC with top 10 BMRGs selected.

**Figure 4:**
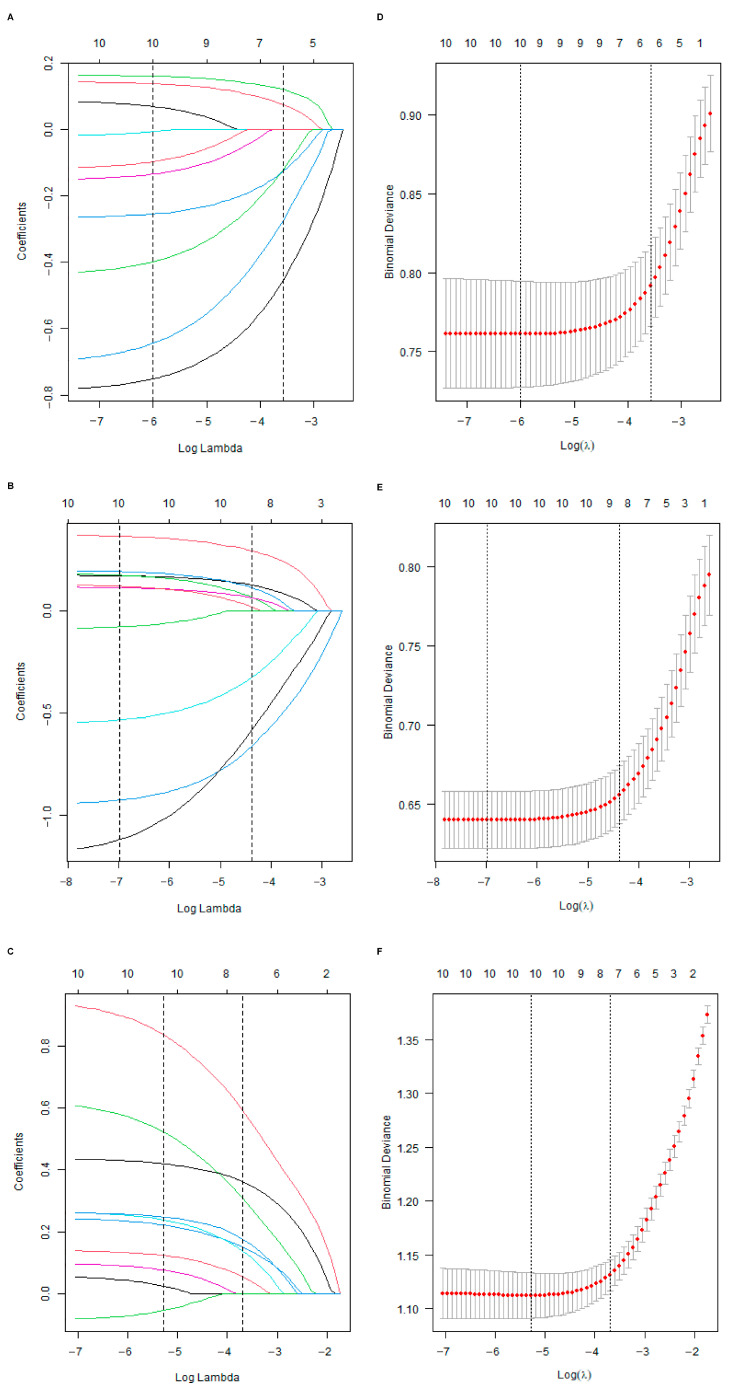
Lasso coefficient model construction. Lasso coefficient model of cross-validation hub genes in (**A**) AK vs. BD, (**B**) AK vs. cSCC, and (**C**) BD vs. cSCC; partial likelihood bias of log-change plotted by Lasso regression of cross-validation in (**D**) AK vs. BD, (**E**) AK vs. cSCC, and (**F**) BD vs. cSCC. Each colored solid line indicates the changing coefficient value of one variable; dashed lines mark specific, chosen λ values.

**Figure 5:**
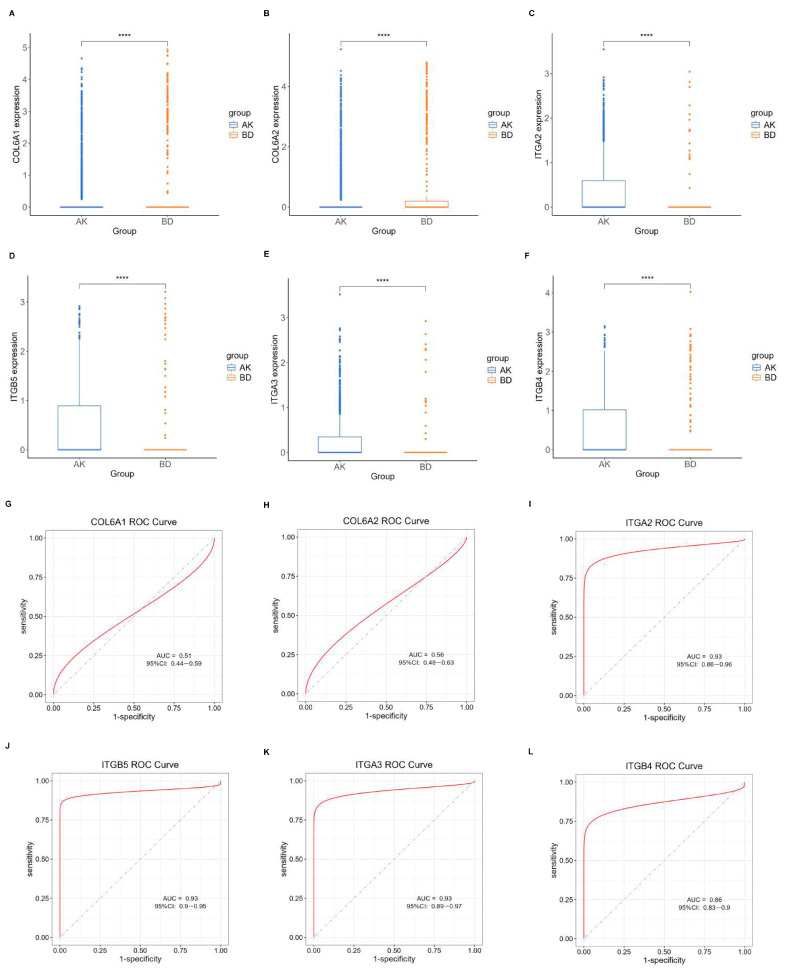
Expression profile of central hub BMRGs in AK vs. BD of keratinocyte clusters. (**A**–**F**) Boxplots of 6 hub genes between AK and BD group; (**G**–**L**) ROC curves of 6 hub genes between AK and BD group. Red curve means the performance trajectory of the model; dashed line indicates the baseline for evaluation. **** adjusted *p* ≤ 0.0001.

Initially, the differential BMRGs were input into the String online database with a standard interaction score of the network set at >0.9 to create PPI network diagrams, and calculations were performed using the CytoHubba (v0.1) plugin in Cytoscape to obtain the top 10 BMRGs (Figure 3 and Figure S3A–C). A Lasso model was constructed on the training set, and the hyperparameter λ was determined through tenfold cross-validation using the minimum criterion. Following the adjustment of parameter selection via cross-validation in Lasso regression, the ultimate central hub BMRGs were acquired for AK vs. BD, AK vs. cSCC, and BD vs. cSCC, respectively (Figure 4 and Figure S3D–I). For keratinocyte clusters, *ITGA2*, *ITGA3*, *ITGB4*, *ITGB5*, *COL6A1*, and *COL6A2* were identified in AK vs. BD; *ITGA2*, *ITGB5*, *ITGB1*, *ITGB8*, *ITGA5*, *ITGA3*, *FN1*, *CD44*, and *LAMA3* in AK vs. cSCC; and *LAMB1*, *LAMC2*, *LAMA3*, *ITGB1*, *ITGA6*, *CD44*, and *SDC1* in BD vs. cSCC. Similarly, for fibroblast clusters, *ITGAV*, *ITGB1*, *LUM*, *BGN*, *FBN1*, *DCN*, and *FN1* were identified in AK vs. BD; *FN1*, *ITGB1*, *LAMB1*, *DCN*, *LAMA2*, and *CD44* in AK vs. cSCC; and *FN1*, *BGN*, *LUM*, *SDC1*, *CD44*, *DCN*, *HSPG2*, *NID1*, *MMP2*, and *FBN1* in BD vs. cSCC (Table 1 and Table S1). ROC curves and AUC statistics were used to validate hub BMRGs, demonstrating their ability to distinguish from healthy controls with high sensitivity and specificity. For AK vs. BD in keratinocyte clusters, the expression of integrin genes was significantly decreased and collagen genes were overexpressed. Most of the BMRGs have a good AUC value (>0.8), indicating the potential for the identification of BD from AK (Figure 5). Similarly, hub genes were significantly differentially expressed in AK vs. cSCC and BD vs. cSCC, with a satisfying AUC value for most BMRGs (Figures S4 and S5). In fibroblast clusters, central hub BMRGs were upregulated or downregulated between each group, showing a good distinctive effect with satisfying AUC values (Figures S6–S8).

### 2.4. Construction and Validation of Nomogram Models

**Figure 6:**
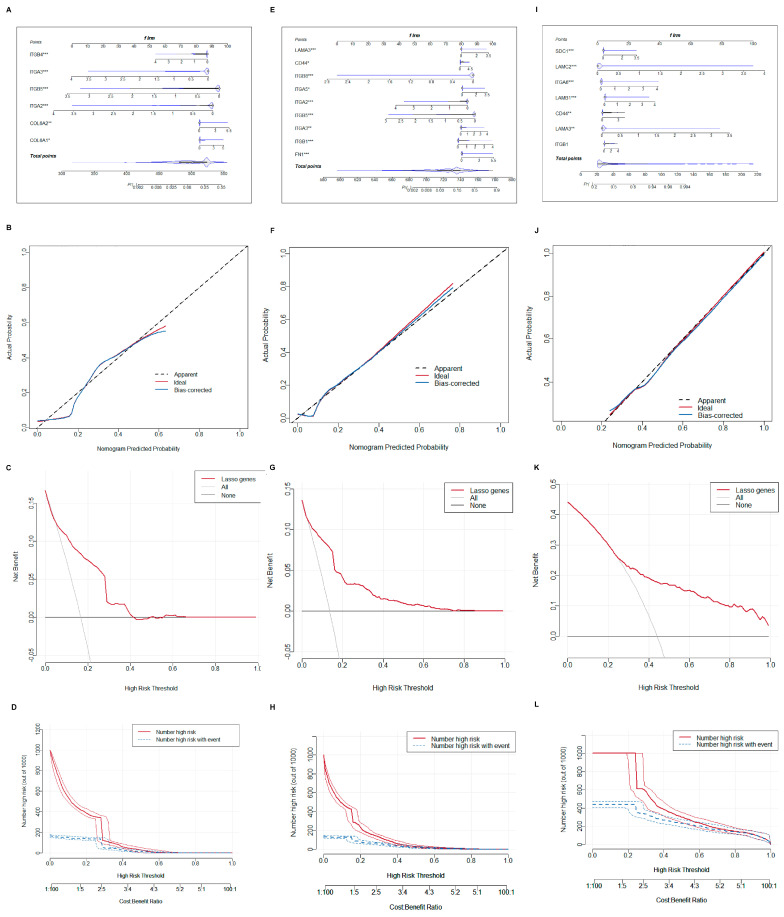
Construction of nomogram model of keratinocyte clusters. (**A**–**D**) Plotting of nomogram, prediction curve, decision curve, and clinical impact curve of the model in AK vs. BD; (**E**–**H**) plotting of nomogram, prediction curve, decision curve, and clinical impact curve of the model in AK vs. cSCC; and (**I**–**L**) plotting of nomogram, prediction curve, decision curve, and clinical impact curve of the model in BD vs. cSCC. * adjusted *p* ≤ 0.05; ** adjusted *p* ≤ 0.01; *** adjusted *p* ≤ 0.001.

To improve diagnostic precision for patients, a nomogram model was built. We selected central hub BMRGs to form a nomogram model for each group of keratinocyte clusters with the predicted curve nearly matching the actual curve, indicating a good distinguishing effect. The model’s accuracy in predicting the occurrence and progression of lesions was confirmed by both the DCA decision curve and the clinical impact curve (Figure 6). Similarly, a nomogram model of fibroblast clusters was also developed with an accurate diagnostic efficient (Figure S9).

### 2.5. Validation of the Central Hub BMRGs

**Figure 7:**
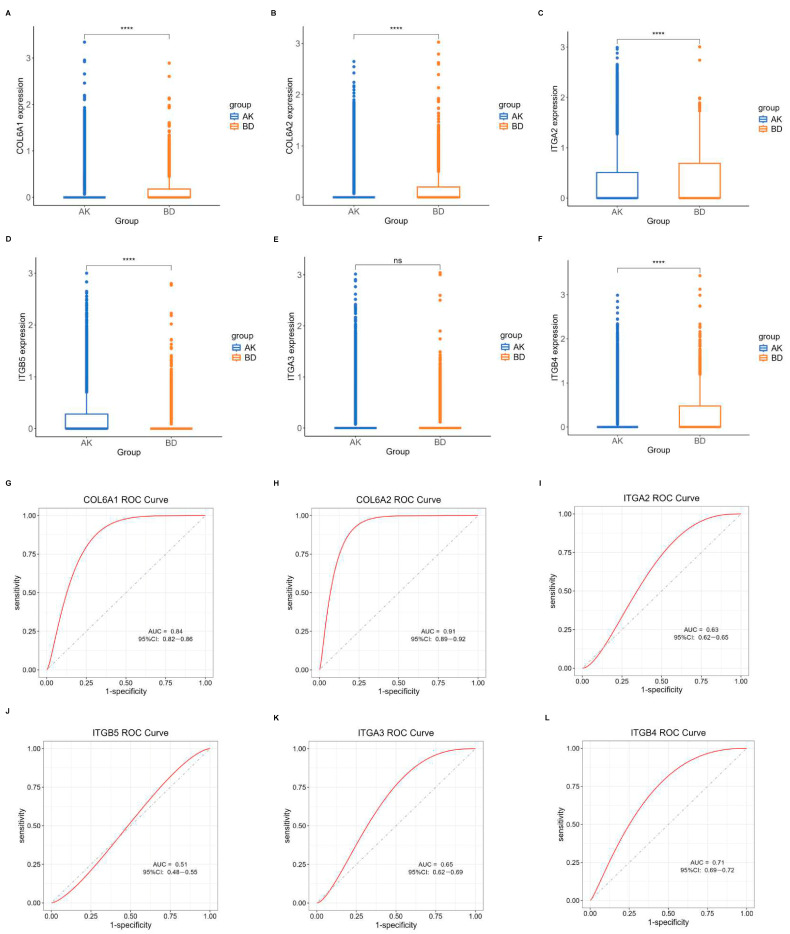
Verification of central hub BMRGs in AK vs. BD of keratinocyte clusters. (**A**–**F**) Boxplots of 6 hub genes between AK and BD group; (**G**–**L**) ROC curves of 6 hub genes between AK and BD group. Red curve means the performance trajectory of the model; dashed line indicates the baseline for evaluation. ns, adjusted *p* > 0.05; **** adjusted *p* ≤ 0.0001.

Another dataset, GSE193304, served to validate the model screening outcomes. The expression levels of the central hub BMRGs in keratinocyte clusters were assessed using boxplots and ROC curves for each group. The predicted BMRGs showed expression differences that aligned with the training group, demonstrating the diagnostic model’s precision and effectiveness (Figure 7). Similarly, central hub BMRGs were also verified with good diagnostic efficiency in validation datasets in other groups and fibroblast clusters (Figures S10–S14). However, some of the genes (e.g., *LAMA3*, *LAMB1*, *ITGA6*, *ITGB1*, *ITGB5* in keratinocytes; *BGN*, *LAMB1* in fibroblasts) were not statistically significant; some inconsistencies in the dataset validation might have resulted from batch variability and diverse sample sources, but these had minimal impact on the overall study.

## 3. Discussion

Cutaneous squamous cell carcinoma poses a significant burden on the health care system with increasing rates of morbidity and mortality [4]. More intensive treatment is needed for invasive cSCC compared to its precursors, such as AK and BD. Surgical excision remains the primary treatment, with adjuvant radiation therapy recommended for high-risk cases, while chemotherapy and targeted therapies are employed for advanced cSCC [1]. However, distinguishing invasive cSCC from pre-invasive lesions like AK or BD poses a challenge. As a hallmark event, morphological breach of BM is a key clue to assessing the invasion of tumors [28]. In this study, we explored, for the first time, a group of novel genes related to basement membrane at a single-cell level to effectively distinguish invasive from pre-invasive lesions. The selected BMRG biomarkers demonstrated significant capability in identifying patients at different stages of cSCC progression, as well as some being capable of indicating different transformation pathways from AK or BD to cSCC.

Among keratinocytes, some of the identified BMRGs have been reported to promote tumorigenesis in other squamous cell carcinomas [29,30]. Although cSCC can progress from AK or BD, there is limited biological evidence to differentiate these two pathways [12]. In this study, the differential expression of BMRGs was identified between cSCC and AK or BD. Compared to AK, integrins (*ITGB1*, *ITGA3*, *ITGA6*), laminins (*LAMA3*, *LAMC1*), *CD44*, and *FN1* were all upregulated in cSCC. Compared to BD, integrins (*ITGB1*, *ITGA3*, *ITGA6*), laminins (*LAMA3*, *LAMB1*, *LAMC1*, *LAMC2*), and *CD44* were upregulated while *FN1* was downregulated in cSCC. The BM and dense fibrotic ECM provided a physical barrier that retained tumor cells at their origin [31,32,33], and integrins played diverse roles in overcoming these barriers through coordinating proteolytic degradation of the BM and stromal ECM, and by enabling migration in complex ECM landscapes [31,34]. Integrins are cell surface receptors that can bind to various ECM proteins, including laminins and fibronectin (FN1) [35,36]. Integrins, particularly the β1-containing integrins, can play a crucial role in mediating cell–ECM interactions and promoting cancer cell migration, invasion, and metastasis [37,38]. However, integrin-mediated EMT regulation is highly variable between different cancer types. *ITGB1* and *ITGA6* were reported to be involved in the progression and metastasis of laryngeal squamous cell carcinoma, esophageal squamous cell carcinoma, oral squamous cell carcinoma, and melanomas [39,40,41]. *ITGB1* has also been shown to mediate invasion of E-cadherin-impaired carcinomas [42], suggesting that the breach of BM may be related to the overexpression of these genes. CD44, selectively activated by hyaluronan, is a classical prognostic factor in various epithelial tumors [43], and its overexpression appears to confer malignant properties to cSCC [44,45]. Laminins are extracellular matrix (ECM) components that are localized around blood vessels and can serve as important signaling molecules for cancer cells [46]. Laminins can interact with integrin receptors on the cell surface, which can promote tumor progression and metastasis [37,38]. And the laminin family, including *LAMC1*, *LAMC2*, and *LAMA3*, may participate in the keratinocyte migration and the aggressive behavior of cSCC [47], having been identified as therapeutic or prognostic biomarkers in multiple squamous cell carcinoma and adenocarcinoma as well [48,49,50]. FN1 is a key ECM component that can interact with various integrin receptors and promote tumor progression through mechanisms such as interfering with immune function or chemotherapy resistance [35]. In our study, *FN1* was identified as a distinct gene of cSCC compared to AK or BD, which tended to be upregulated in creating a pro-metastatic environment for cancer cells in conjunction with integrins [51]. FN1 could also guide the collective migration of carcinoma cells and was differentially expressed in signaling pathways related to cancer progression [37,52]. Therefore, BMRGs of the integrin and laminin family may be considered attractive drug targets in skin cancer.

Among fibroblasts, *ITGB1*, *ITGAV*, *FN1*, *BGN*, *SDC1*, and *LUM* were all upregulated in cSCC, while *CD44* and *DCN* were downregulated compared to AK. Compared to BD, *FN1*, *BGN*, *SDC1*, and *LUM* were also upregulated genes in cSCC; however, in addition to *CD44* and *DCN*, the downregulated BMRGs included *HSPG2*, *MMP2*, *NID1*, etc. There was a notable difference between these two groups. Fibroblasts, particularly cancer-associated fibroblasts, were believed to interact with and pull the basement membrane, likely via integrin-mediated adhesion, forming gaps that cancer cells could move through [53]. The BM offered structural support and acted as a physical barrier between the epithelial and stromal areas, which could be compromised by the proteolytic breakdown of the ECM by MMPs and often with an increase of invasive cancer cells [20]. The collaboration between MMP and integrins has been documented in various carcinomas, where increased MMP levels often boost cancer cell invasion. Elevated levels of ITGA3 and MMP3 have been linked to lower survival rates in patients with squamous cell carcinoma [54]. The cleavage of fibronectin by MMP2 can result in increased melanoma cell migration facilitated by ITGAV/B3 [55]. SDC1 is a cell-surface heparan sulfate proteoglycan involved in cell proliferation, tumor invasion or progression of various squamous cell carcinoma, and adenocarcinoma, especially the cSCC [56,57,58,59]. Several studies indicated that higher *BGN*/lower *DCN* level showed poor overall survival in head and neck SCC, as well as oral squamous cell carcinomas [60,61]. The expression pattern of these genes in fibroblast is similar to previous studies, which may also suggest a poor outcome for cSCC. Impressively, *CD44*, identified in fibroblast clusters, was downregulated in cSCC, exhibiting a different expression pattern compared to that of keratinocytes. In fact, downregulation of *CD44* has been proven to be associate with poor prognosis in certain tumors like oral squamous cell carcinomas [62]. In addition, as a BMRG, *NID1* was dramatically identified to be upregulated in cSCC compared to AK, but downregulated in cSCC compared to BD. Previous studies have also shown decreased expression of *NID1* in cSCC but overexpression in basal cell carcinoma, which has been consistent with our study [63]. Therefore, these BMRGs may serve as potential biomarkers in fibroblast clusters to identify malignancy and BM breach.

Although both BD and progressing AK are cSCC in situ, their etiology and prognostic risks are different. Cumulative exposure to sunlight is an important factor in AK carcinogenesis, whereas solar radiation, arsenic ingestion, and human papillomavirus infection are implicated in the etiology of BD. For example, Bowenoid AK and BD in sun-exposed areas are often difficult to distinguish. Among keratinocytes, integrins (*ITGB1*, *ITGA6*, *ITGAV*), and collagens (*COL6A1*, *COL6A2*) were all upregulated in BD compared to AK, indicating a higher risk of BM breach and invasive potential for BD lesions [64]. Among fibroblasts, other than some overexpressed integrin genes like *ITGB1* and *ITGAV*, other genes like *LUM*, *BGN*, and *SDC1* were also upregulated in BD together with downregulation of *DCN* and *FN1*. *LUM*, which belongs to the family of small leucine-rich repeat proteoglycans, has been identified and shown potential in differentially diagnosing AK and BD in previous studies [65]. *LUM* is localized in cancer cells and the fibroblasts adjacent to them, with expression observed in BD but not in AK. Thus, the distinct expression patterns of *LUM* in AK and BD might explain the different pathogenesis and progression risk of these two diseases.

Although this study has predicted several key differentially expressed basement membrane genes, it remains unclear whether they truly exhibit differential expression in cutaneous squamous cell carcinoma. Therefore, further in vitro and in vivo experiments are needed for a comprehensive evaluation, and related studies should be designed to explore their carcinogenic or tumor-suppressive mechanisms. Additionally, both our training and validation cohorts were relatively small, leading to somewhat insufficient supporting evidence. Moving forward, we will continue to monitor updates in relevant databases or collect clinical samples independently to expand the sample size and further validate the findings.

## 4. Materials and Methods

### 4.1. Data Sources

Multiple gene expression profile datasets were searched from GEO datasets in our study, including GSE218170 (3 samples of healthy skin tissues, 3 samples of BD, and 3 samples of cSCC) (https://www.ncbi.nlm.nih.gov/geo/query/acc.cgi?acc=GSE218170, accessed on 21 January 2025), GSE200334 (3 samples of AK) (https://www.ncbi.nlm.nih.gov/geo/query/acc.cgi?acc=GSE200334, accessed on 21 January 2025), and GSE193304 (6 samples of healthy skin tissues, 3 samples of AK, 1 samples of BD, and 3 samples of cSCC) (https://www.ncbi.nlm.nih.gov/geo/query/acc.cgi?acc=GSE193304, accessed on 21 January 2025). The first two datasets served as the training sets and the last one was used for validation analysis. Single-cell RNA transcriptomic profiles of all samples were downloaded for subsequent analysis. To identify BMRGs, we referred to a previously published report that comprehensively delineated a network of 222 human proteins and their animal orthologs related to BMs [24].

### 4.2. Identification of Differentially Expressed BMRGs

10X Genomics Cell Ranger software (version 3.1.0, Pleasanton, CA, USA) was used to convert raw BCL files to FASTQ files, as well as alignment and counts quantification [66]. Batch effects were adjusted and principal component analysis was performed, followed by cell clustering, visualization, and annotation. Differentially expressed genes (DEGs) analysis in a given cluster were compared against the rest of cells using Wilcoxon rank sum test. *p* values were adjusted for multiple testing using the Benjamini–Hochberg (BH) method to control the false discovery rate (FDR). Genes with an absolute log fold change (|logFC|) greater than 0.5 and an adjusted *p* value < 0.05 were considered statistically significant [67]. Finally, differentially expressed BMRGs were identified between AK and BD, AK and cSCC, and BD and cSCC, respectively, in keratinocyte and fibroblast clusters and then visualized via heatmaps and volcano plots.

### 4.3. Functional Enrichment Analysis

Functional gene annotation was performed using Gene Ontology (GO), covering molecular function (MF), biological process (BP), and cellular component (CC) annotations. Kyoto encyclopedia of genes and genomes (KEGG) enrichment analysis served as a valuable reference for functional research on DEGs [68]. A two-tailed Fisher’s exact test was used to test the GO and KEGG enrichment of the DEGs against all identified genes, and an adjusted *p* < 0.05 was considered significant.

### 4.4. Identification of Hub BMRGs

Hub differentially expressed BMRGs were further screened between each group. First, we selected significantly differentially expressed BMRGs from all DEGs as targeted gene sets. A protein-protein interaction (PPI) network was constructed with a minimum interaction distance of 0.9. Cytoscape (version 3.9.1, open-source, Toronto, ON, Canada) was then used to visualize the network model and employed CytoHubba to identify the top 10 BMRGs [69]. Furthermore, we performed least absolute shrinkage and selection operator (Lasso) regression in order to compute linear models and screen for the central hub BMRGs. The central hub BMRG expression levels between each group were shown using boxplots, with the ROC plots drawn and the AUCs determined.

### 4.5. Nomogram Model Design and Validation

The ‘regplot’ R package (version 1.1, open-source, The Comprehensive R Archive Network) was used to construct and display nomograms [70]. Subsequently, calibration graphs were created to evaluate the accuracy of the nomogram, and decision curve analysis (DCA) was conducted to examine the net clinical advantage of the nomogram using the R packages ‘caret’ and ‘rmda’. The level of distinction of central hub genes across each group was assessed.

### 4.6. Statistical Analysis

All the statistical analyses were completed on R4.1.0 (open-source, R Foundation for Statistical Computing, Vienna, Austria). The correlations between variables were analyzed using Pearson and Spearman methods. Comparisons between different subgroup samples were performed using nonpara-metric Wilcoxon rank sum test. *p* values were adjusted for multiple testing using the BH method, and an adjusted *p* value < 0.05 was considered statistically significant. The statistical significance was denoted as follows: ns, adjusted *p* > 0.05; * adjusted *p* ≤ 0.05; ** adjusted *p* ≤ 0.01; *** adjusted *p* ≤ 0.001; and **** adjusted *p* ≤ 0.0001.

## 5. Conclusions

In this study, multiple BMRG biomarkers were identified based on two main cell clusters: keratinocytes and fibroblasts. Within each cell subgroup, upregulated or downregulated hub BMRGs were identified to distinguish cSCC from AK or BD, revealing genetic characteristics of the two distinct progression pathways of cSCC. Furthermore, novel biological evidence was selected to support the distinction between AK and BD. The main innovation of this manuscript lies in exploring several hub BMRGs, such as laminins, integrins, *LUM*, *FN1*, and so on, in the progression and transformation of cSCC using scRNA-seq. Further investigation about the interplay within these BMRGs in the tumor microenvironment is needed, which may be potential targets for therapeutic intervention against cSCC and pre-invasive lesions in the future.

## Figures and Tables

**Table 1:** Hub BMRGs of keratinocyte clusters.

Rank	AK vs. BD		AK vs. cSCC		BD vs. cSCC	
	PPI	Lasso	PPI	Lasso	PPI	Lasso
1	*ITGB1*	*-*	*FN1*	*FN1*	*FN1*	*-*
2	*COL6A1*	*COL6A1*	*ITGB1*	*ITGB1*	*ITGB1*	*ITGB1*
3	*COL6A2*	*COL6A2*	*ITGA3*	*ITGA3*	*LAMA3*	*LAMA3*
4	*ITGA2*	*ITGA2*	*ITGB5*	*ITGB5*	*CD44*	*CD44*
5	*ITGA6*	*-*	*ITGA2*	*ITGA2*	*LAMB1*	*LAMB1*
6	*ITGAV*	*-*	*ITGA5*	*ITGA5*	*LAMC1*	*-*
7	*ITGB5*	*ITGB5*	*ITGB8*	*ITGB8*	*ITGA6*	*ITGA6*
8	*LAMC1*	*-*	*CD44*	*CD44*	*LAMC2*	*LAMC2*
9	*ITGA3*	*ITGA3*	*HSPG2*	*-*	*DCN*	*-*
10	*ITGB4*	*ITGB4*	*LAMA3*	*LAMA3*	*SDC1*	*SDC1*

## Data Availability

The original data presented in the study are openly available in https://www.ncbi.nlm.nih.gov/geo.

## Supplementary Materials

The following supporting information can be downloaded at: https://www.mdpi.com/article/10.3390/ijms27031394/s1.

## Abbreviations

AK	actinic keratosis
BD	Bowen’s disease
BM	basement membrane
BMRGs	BM-related genes
BP	biological process
CAFs	cancer-associated fibroblasts
CC	cellular component
cSCC	cutaneous squamous cell carcinoma
DCA	decision curve analysis
DEGs	differentially expressed genes
ECM	extracellular matrix
GO	Gene Ontology
KEGG	Kyoto encyclopedia of genes and genomes
MF	molecular function
MMPs	matrix metalloproteinases
PCA	principal component analysis
PPI	protein–protein interaction
scRNA-seq	single-cell RNA sequencing
UMAP	uniform manifold approximation and projection
