# Supplementary material for: Identification of Basement Membrane-Related Biomarkers in the Progression of Cutaneous Squamous Cell Carcinoma
Source: Int J Mol Sci. 2026 Jan 30;27(3):1394. doi: 10.3390/ijms27031394 (PMC12898604; doi:10.3390/ijms27031394)
Supplement: Supplementary file 1 [file ijms-27-01394-s001.zip › ijms-4072226-supplementary.pdf]

Supplementary Materials

Identification of Basement Membrane-Related Biomarkers in the Progression of Cutaneous Squamous Cell Carcinoma

Shuaijun Zou [1,†], Sijia Huang [2,†], Jun Liu [3], Ruiqian Yao [1], Xiaoyan Yang [1], Haixia Zhao [1], Lin Du [1], Liangzhe Wang [1,*] and Yuanjie Zhu [1,*]

1 Department of Dermatology, Naval Medical Centre, Naval Medical University, Shanghai 200052, China; smmuzsj@163.com (S.Z.); yaorq999@163.com (R.Y.); xiaoyanyh@163.com (X.Y.); 18101823621@163.com (H.Z.); lynnie_du@126.com (L.D.)
2 School of Medicine, Shanghai University, Shanghai 200444, China; sijia-huang@shu.edu.cn
3 Department of Naval Diving Medicine, Naval Medical Centre, Naval Medical University, Shanghai 200052, China; liujun4531@126.com
* Correspondence: lzwang@hotmail.com (L.W.); zhuyj@smmu.edu.cn (Y.Z.)
† These authors contributed equally to this work.

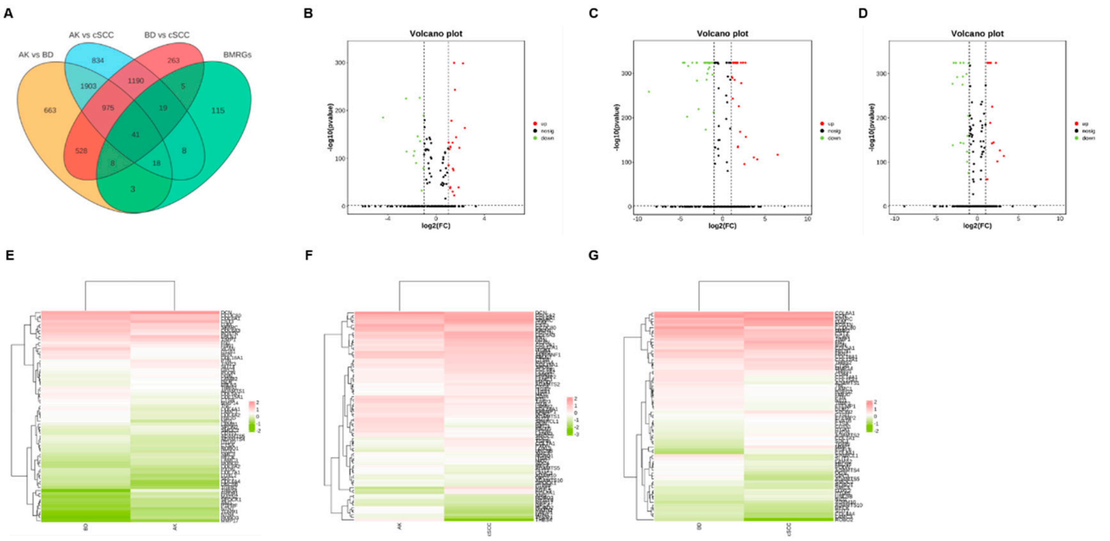

Figure S1. Differentially expressed BMRGs of fibroblast clusters. (A) Venn diagram showing DEGs and differentially expressed BNRGs in fibroblast clusters; Volcano plots of BMRGs in **(B)** AK *vs* BD, **(C)** AK *vs* cSCC and **(D)** BD *vs* cSCC; Heatmap of BMRGs in **(E)** AK *vs* BD, **(F)** AK *vs* cSCC and **(G)** BD *vs* cSCC.

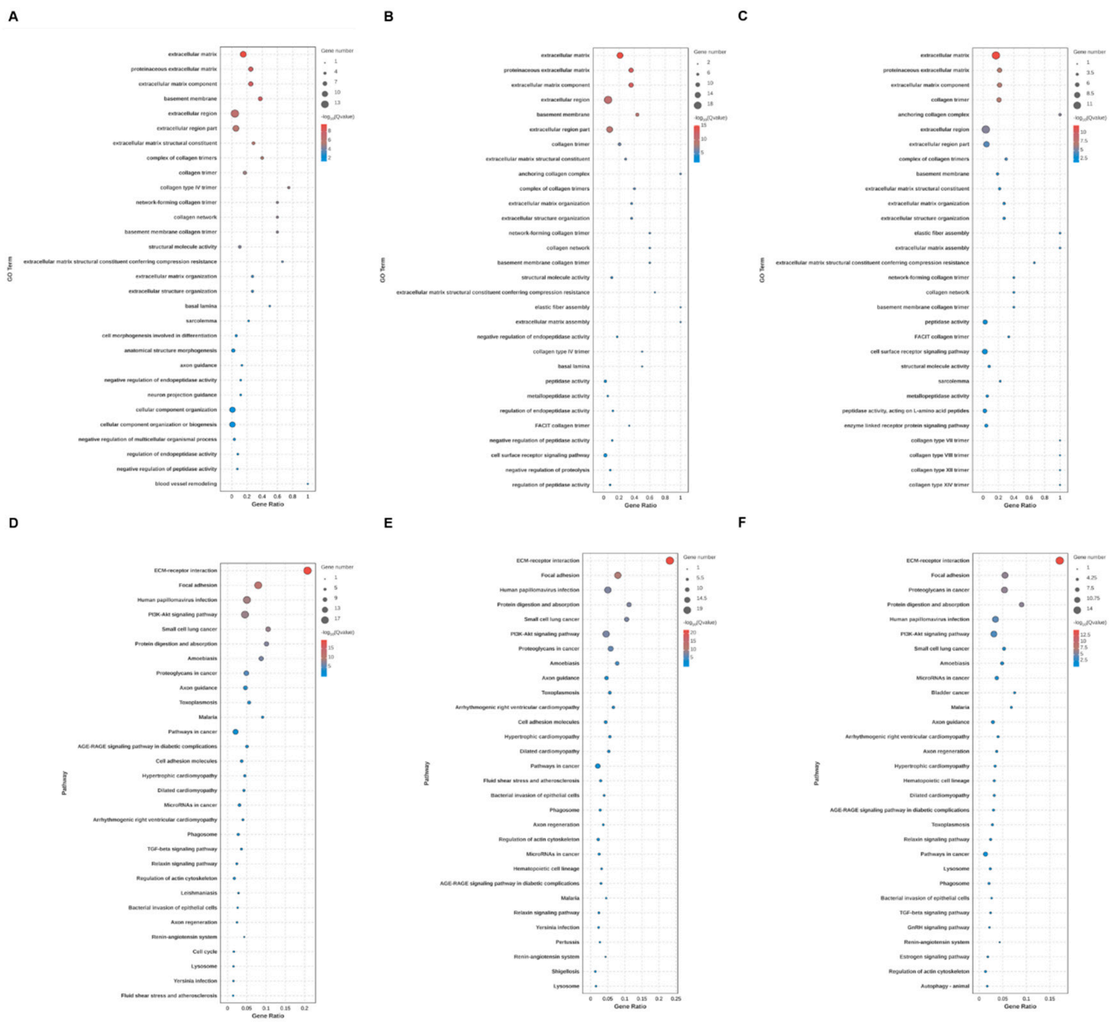

Figure S2. Functional enrichment analysis of fibroblast clusters. (A)~(B) Bubble chart of GO enrichment analysis and KEGG enrichment analysis of AK *vs* BD with top 30 functions or pathways listed. **(C)~(D)** Bubble chart of GO enrichment analysis and KEGG enrichment analysis of AK *vs* cSCC with top 30 functions or pathways listed. **(E)~(F)** Bubble chart of GO enrichment analysis and KEGG enrichment analysis of BD *vs* cSCC with top 30 functions or pathways listed.

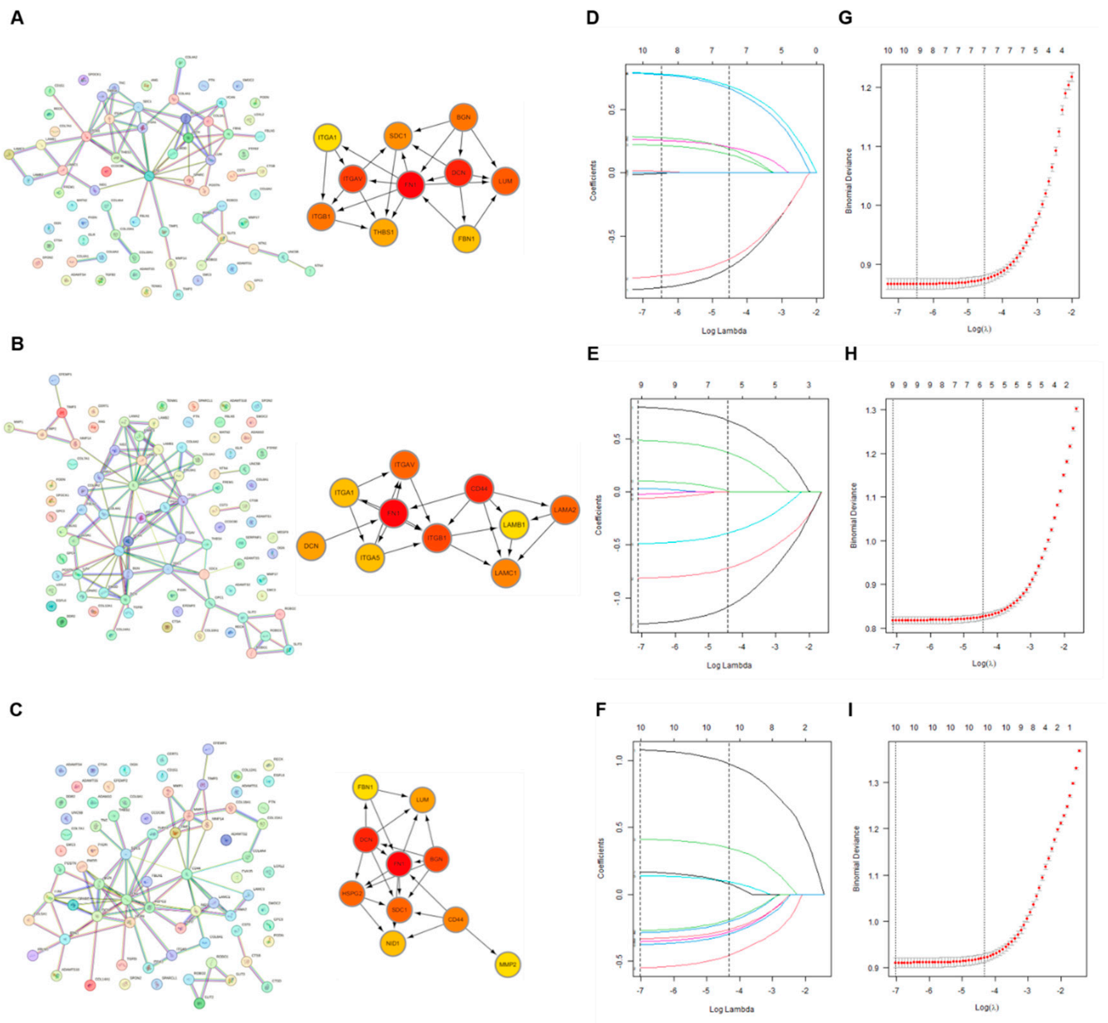

Figure S3. Hub BMRGs screening of fibroblast clusters. PPI network construction of differentiated expressed BMRGs in **(A)**AK *vs* BD, **(B)** AK *vs* cSCC and **(C)** BD *vs* cSCC with top 10 BMRGs selected; Lasso coefficient model of cross-validation hub genes in **(D)**AK *vs* BD, **(E)** AK *vs* cSCC and **(F)** BD *vs* cSCC; Partial likelihood bias of log-change plotted by Lasso regression in cross-validation of **(G)**AK *vs* BD, **(H)** AK *vs* cSCC and **(I)** BD *vs* cSCC.

Table S1. Hub BMRGs of each group in fibroblast clusters.

Rank	AK/BD		AK/cSCC		BD/cSCC	
	**PPI**	**Lasso**	**PPI**	**Lasso**	**PPI**	**Lasso**
1	FN1	FN1	FN1	FN1	FN1	FN1
2	DCN	DCN	CD44	CD44	DCN	DCN
3	ITGAV	ITGAV	ITGB1	ITGB1	BGN	BGN
4	LUM	LUM	ITGAV	-	HSPG2	HSPG2
5	ITGB1	ITGB1	LAMA2	LAMA2	SDC1	SDC1
6	BGN	BGN	LAMC1	-	CD44	CD44
7	SDC1	-	DCN	DCN	LUM	LUM
8	THBS1	-	ITGA1	-	NID1	NID1
9	FBN1	FBN1	ITGA5	-	MMP2	MMP2
10	ITGA1	-	LAMB1	LAMB1	FBN1	FBN1

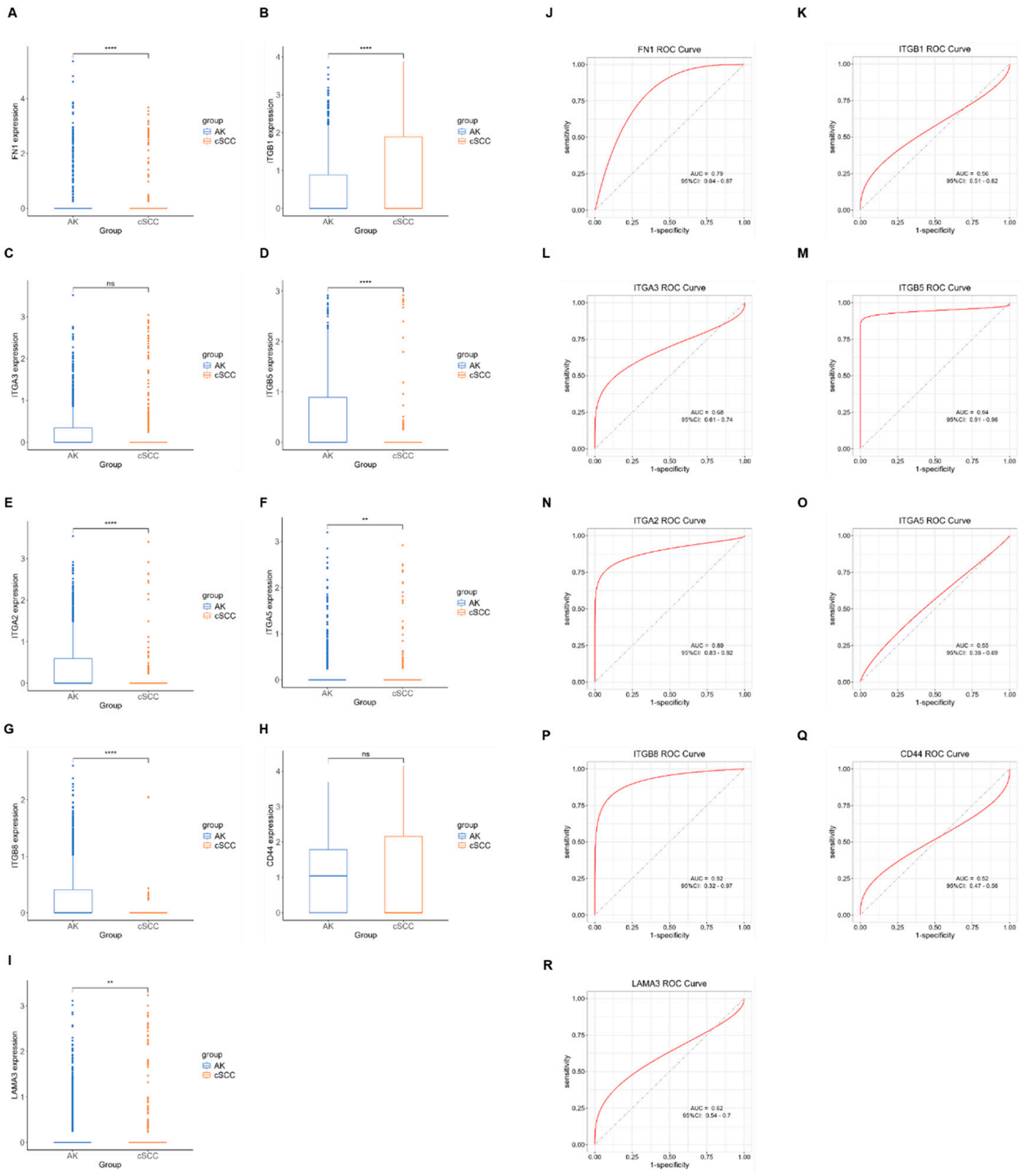

Figure S4. Expression profile of central hub BMRGs in AK *vs* cSCC of keratinocyte clusters. (A)~(I) Boxplots of 9 hub genes between AK and BD group; **(J)~(R)** ROC curves of 9 hub genes between AK and BD group.

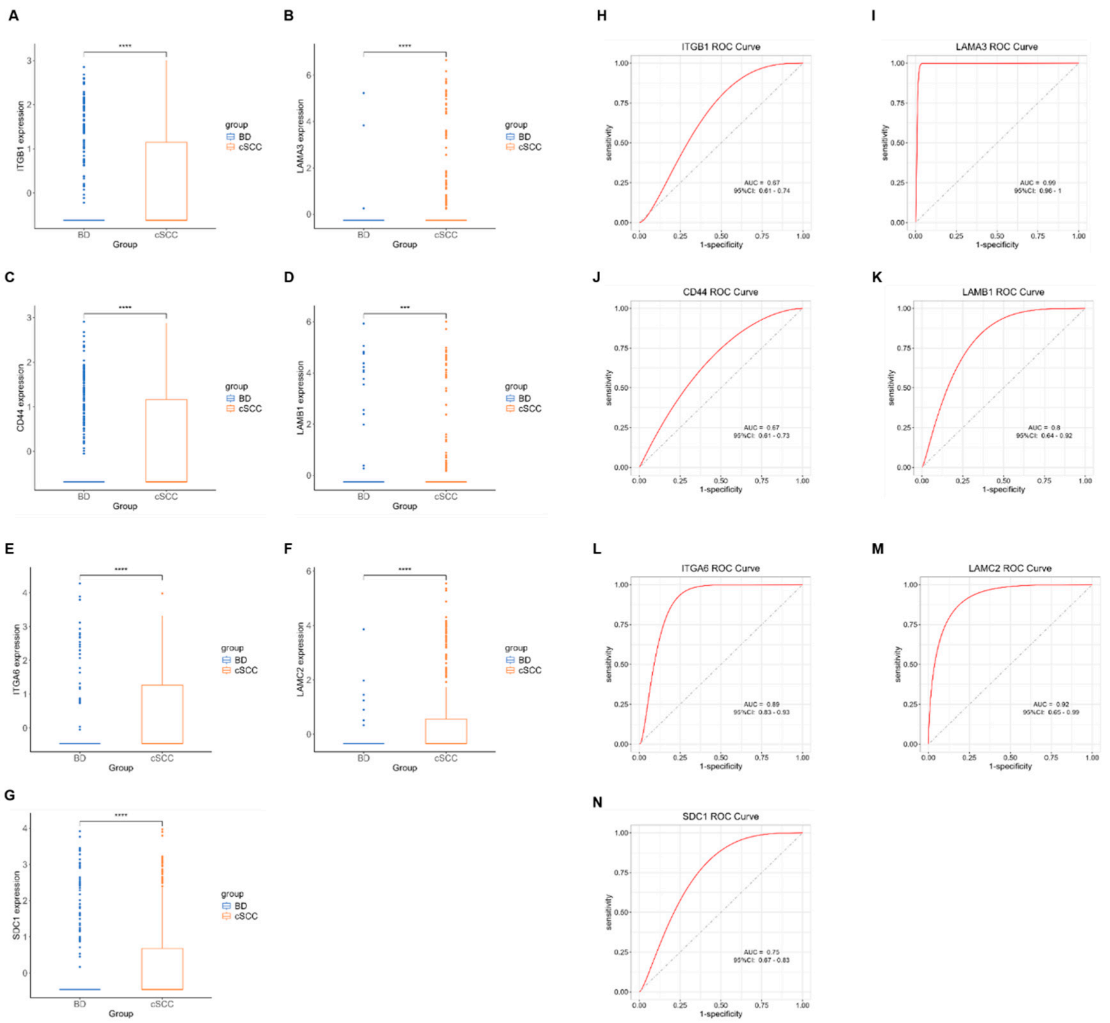

Figure S5. Expression profile of central hub BMRGs in BD *vs* cSCC of keratinocyte clusters. (A)~(G) Boxplots of 7 hub genes between AK and BD group; **(H)~(N)** ROC curves of 7 hub genes between AK and BD group.

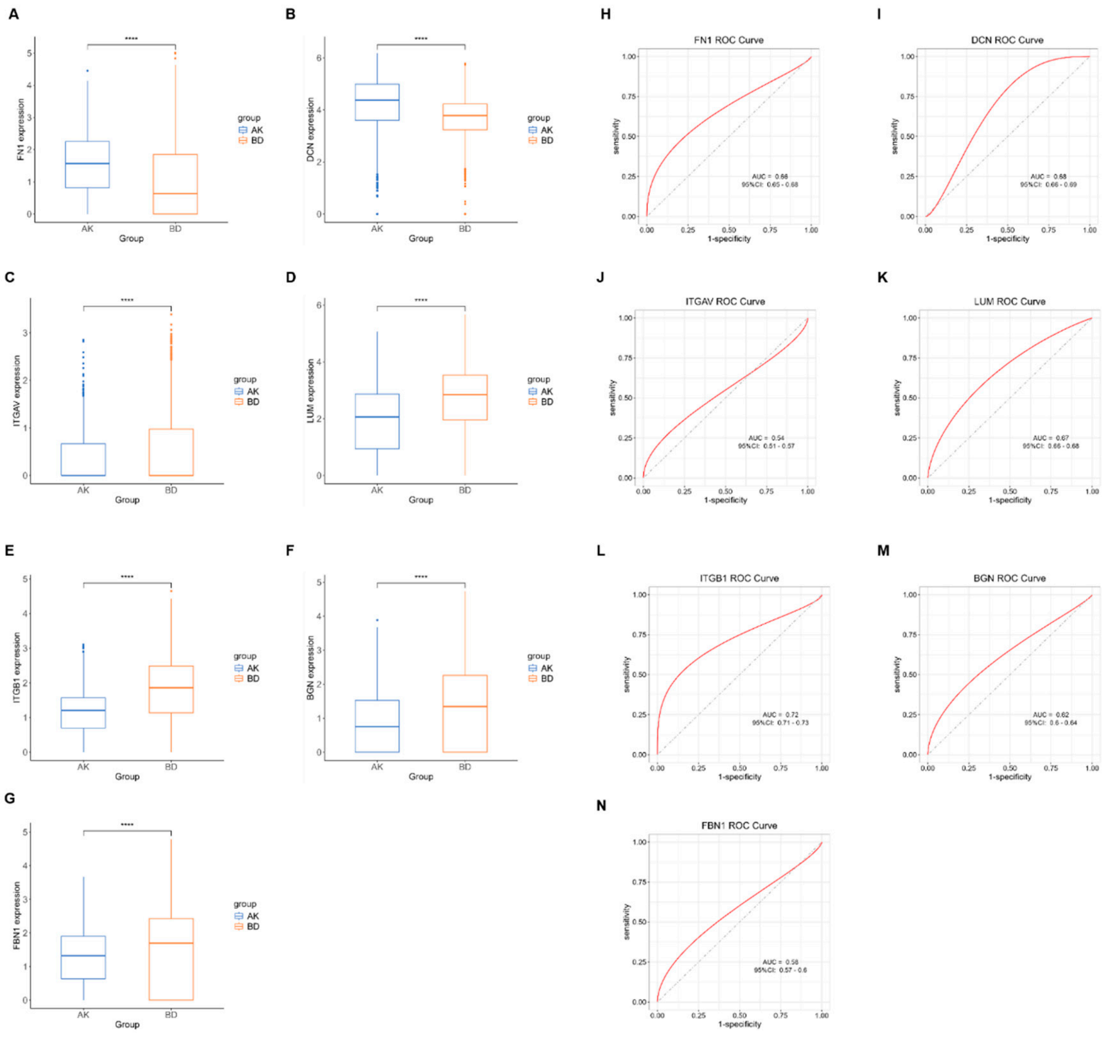

Figure S6. Expression profile of central hub BMRGs in AK *vs* BD of fibroblast clusters. (A)~(G) Boxplots of 7 hub genes between AK and BD group; **(H)~(N)** ROC curves of 7 hub genes between AK and BD group.

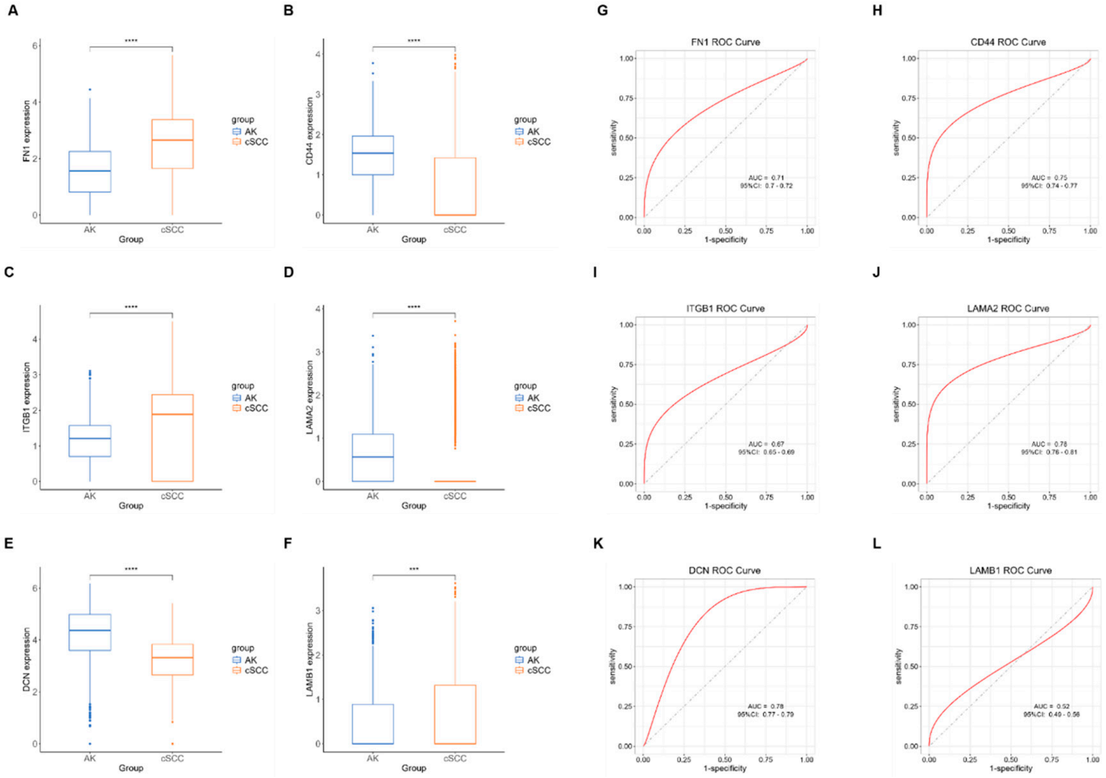

Figure S7. Expression profile of central hub BMRGs in AK *vs* cSCC of fibroblast clusters. (A)~(F) Boxplots of 6 hub genes between AK and BD group; **(G)~(M)** ROC curves of 6 hub genes between AK and BD group.

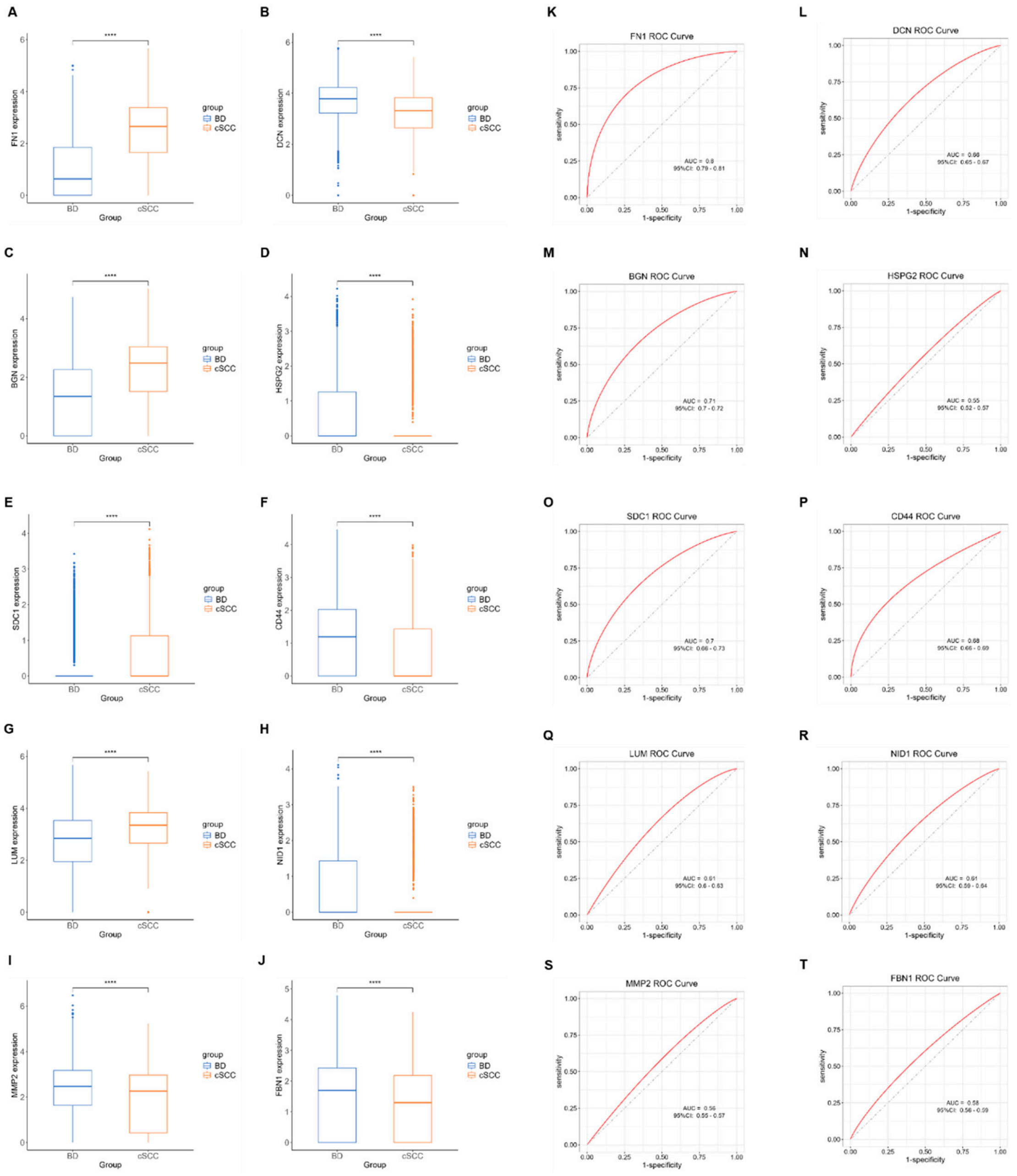

Figure S8. Expression profile of central hub BMRGs in BD *vs* cSCC of fibroblast clusters. (A)~(J) Boxplots of 10 hub genes between AK and BD group; **(K)~(T)** ROC curves of 10 hub genes between AK and BD group.

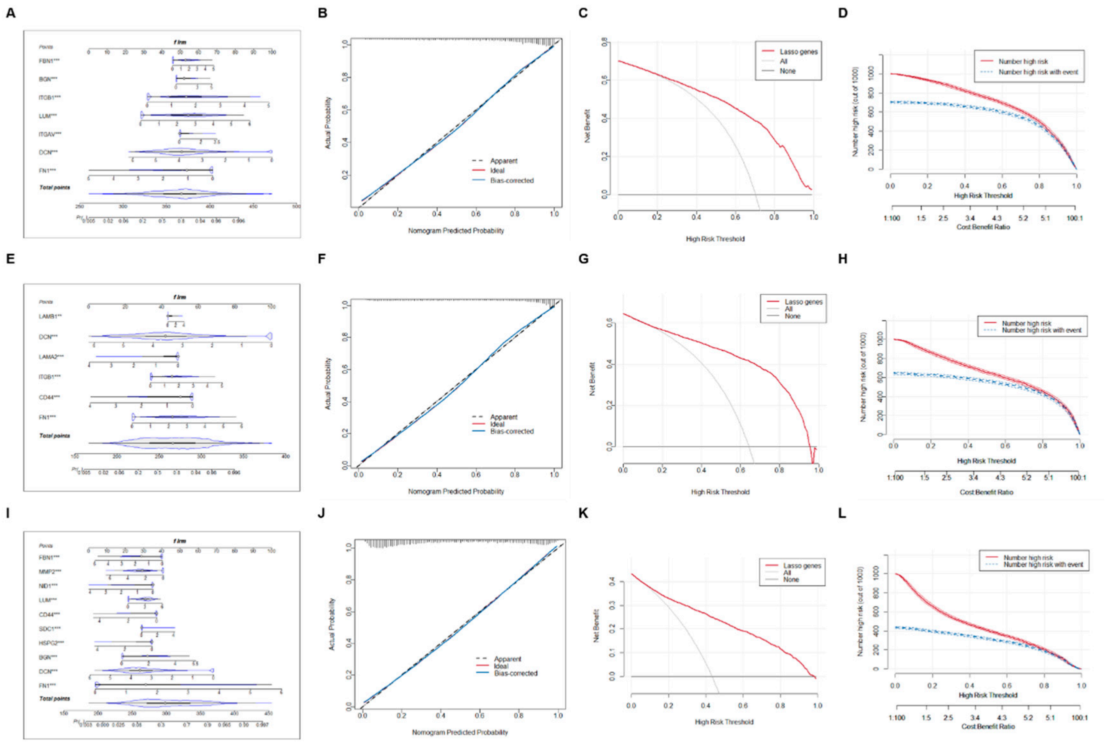

Figure S9. Construction of nomogram model of fibroblast clusters. (A)~(D) Plotting of nomogram, prediction curve, decision curve, and clinical impact curve of the model in AK *vs* BD; **(E)~(H)** Plotting of nomogram, prediction curve, decision curve, and clinical impact curve of the model in AK *vs* cSCC; **(I)~(L)** Plotting of nomogram, prediction curve, decision curve, and clinical impact curve of the model in BD *vs* cSCC.

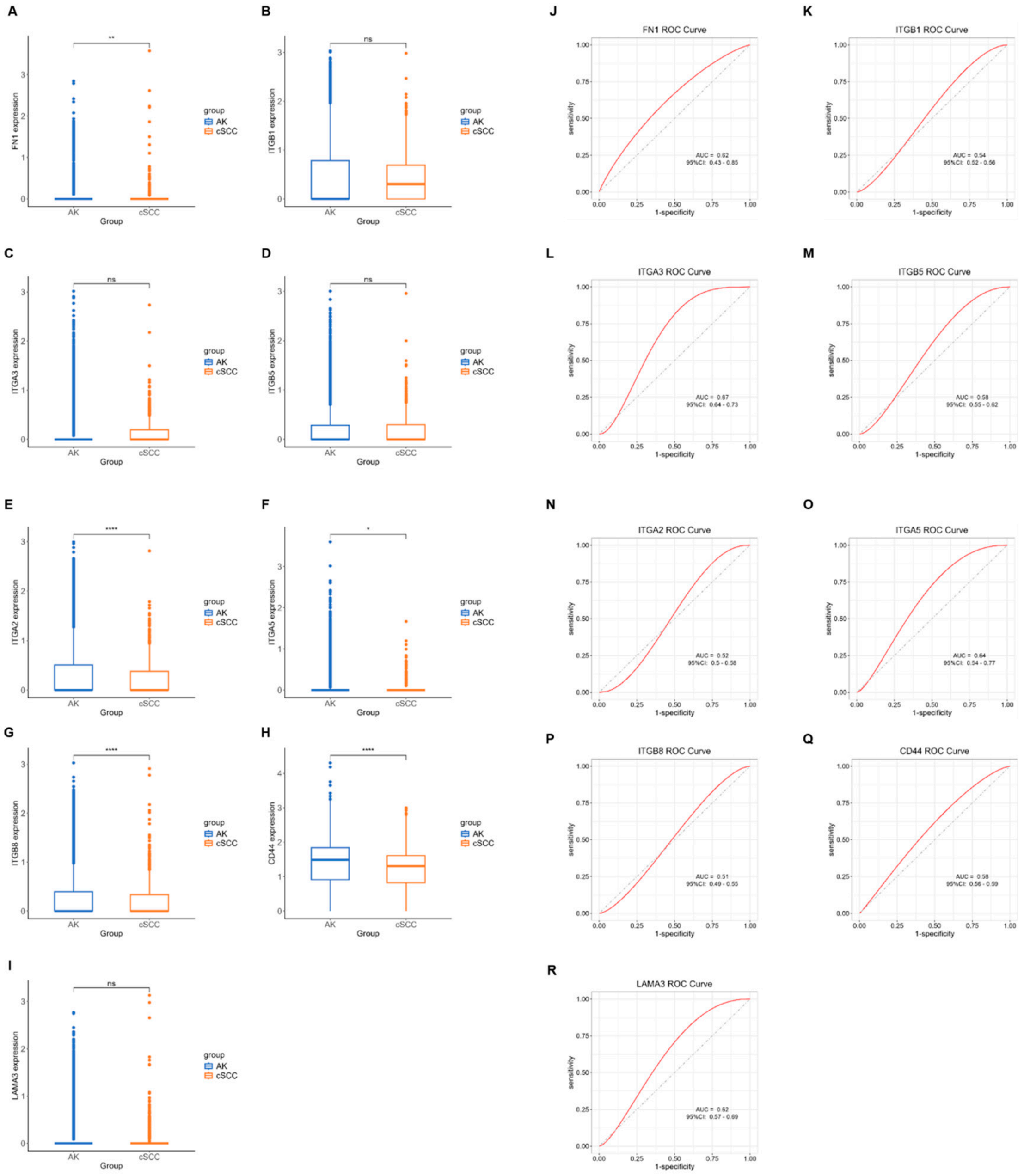

Figure S10. Verification of central hub BMRGs in AK *vs* cSCC of keratinocyte clusters. (A)~(I) Boxplots of 9 hub genes between AK and BD group; **(J)~(R)** ROC curves of 9 hub genes between AK and BD group.

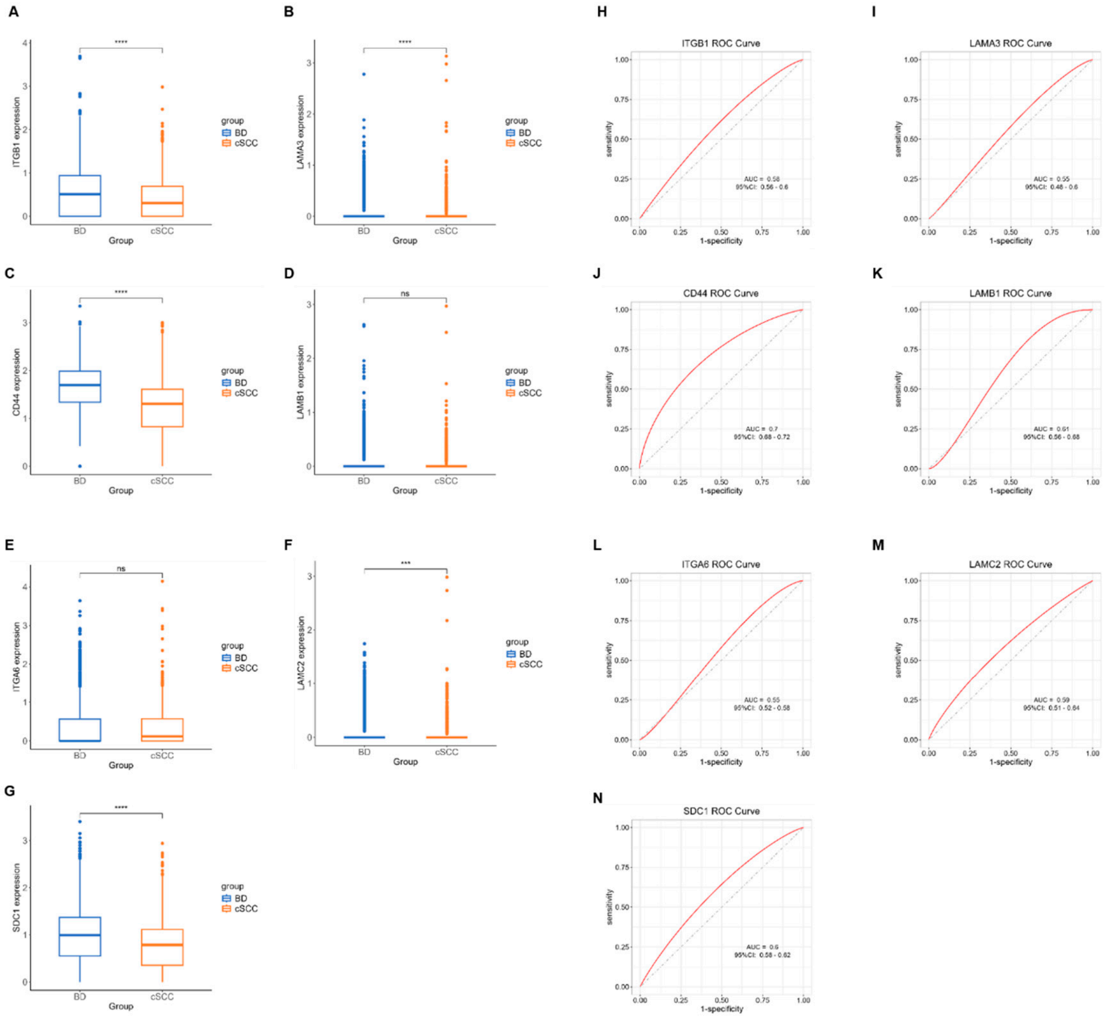

Figure S11. Verification of central hub BMRGs in BD *vs* cSCC of keratinocyte clusters. (A)~(G) Boxplots of 7 hub genes between AK and BD group; **(H)~(N)** ROC curves of 7 hub genes between AK and BD group.

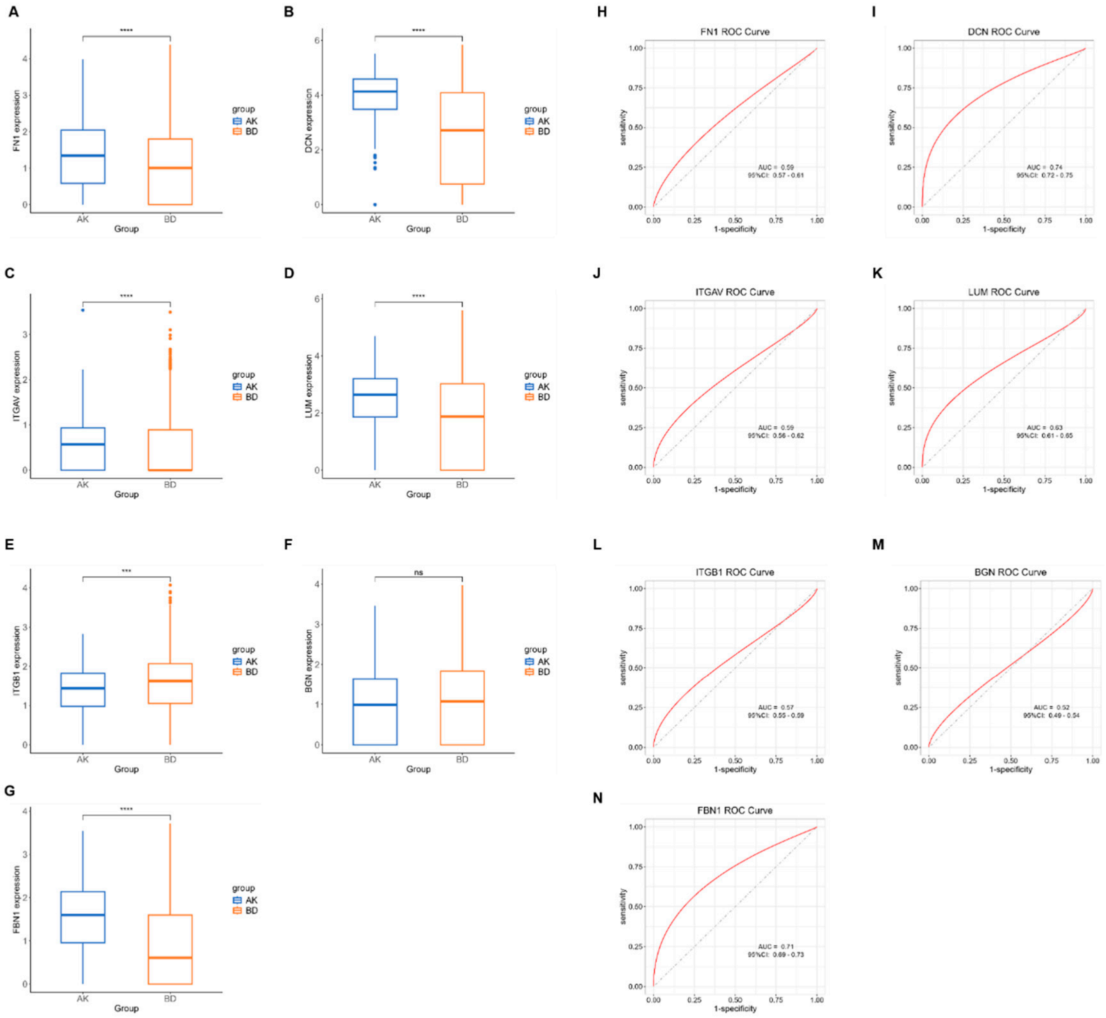

Figure S12. Verification of central hub BMRGs in AK *vs* BD of fibroblast clusters. (A)~(G) Boxplots of 7 hub genes between AK and BD group; **(H)~(N)** ROC curves of 7 hub genes between AK and BD group.

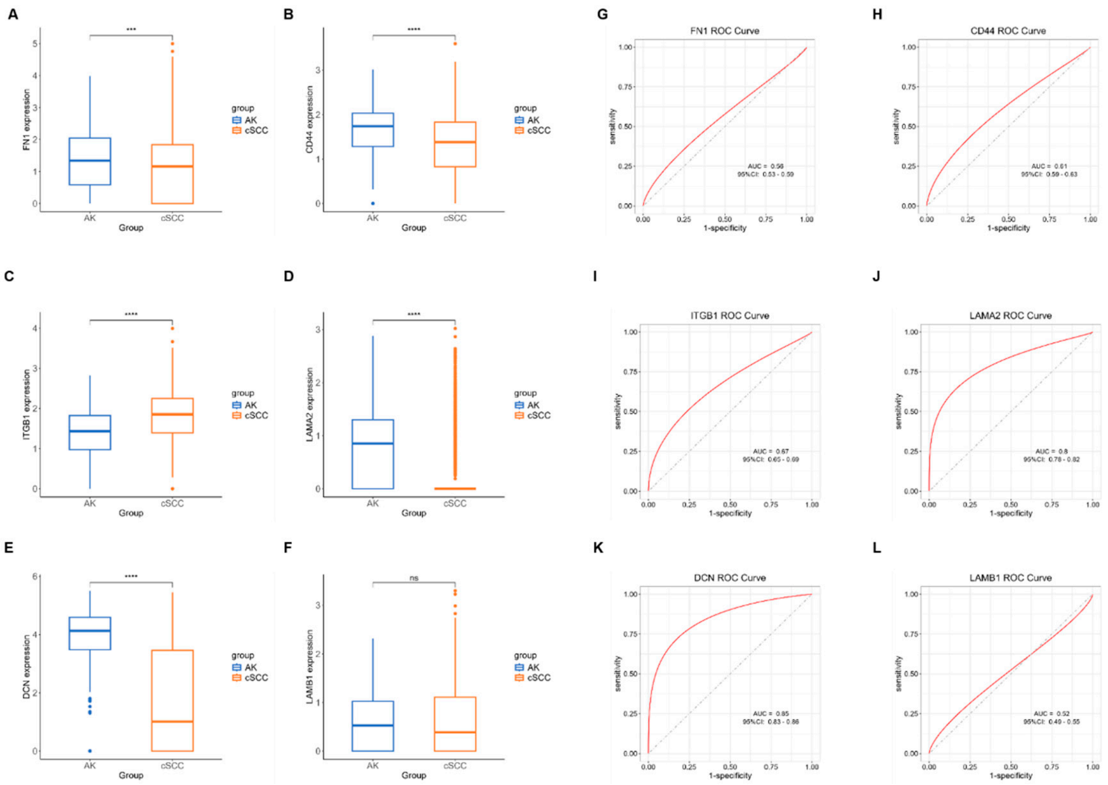

Figure S13. Verification of central hub BMRGs in AK *vs* cSCC of fibroblast clusters. (A)~(F) Boxplots of 6 hub genes between AK and BD group; **(G)~(M)** ROC curves of 6 hub genes between AK and BD group.

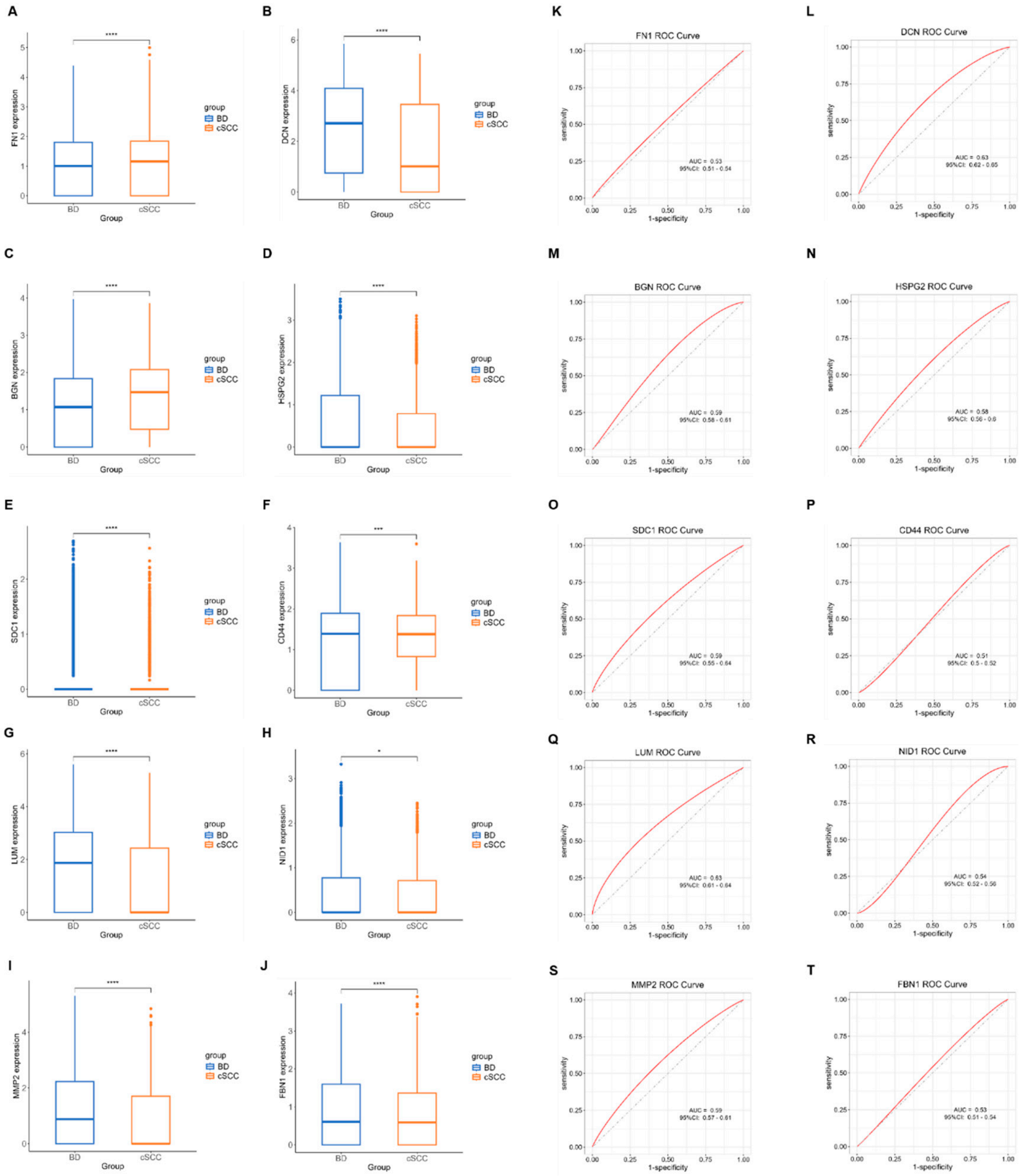

Figure S14. Verification of central hub BMRGs in BD *vs* cSCC of fibroblast clusters. (A)~(J) Boxplots of 10 hub genes between AK and BD group; **(K)~(T)** ROC curves of 10 hub genes between AK and BD group.
